# Risk of 90-day readmission in patients after firearm injury hospitalization: a nationally representative retrospective cohort study

**DOI:** 10.5249/jivr.v11i1.979

**Published:** 2019-01

**Authors:** Bindu Kalesan, Yi Zuo, Ramachandran S. Vasan, Sandro Galea

**Affiliations:** ^*a*^Department of Medicine and Community Health Science, Schools of Medicine and Public Health, Boston University, Boston, MA, USA.; ^*b*^Department of Medicine, School of Medicine, Boston University, Boston, MA, USA.; ^*c*^Departments of Medicine and Epidemiology, Schools of Medicine and Public Health, Boston University, Boston, MA, USA.; ^*d*^Dean’s Office, School of Public Health, Boston University, Boston, MA, USA.

**Keywords:** Firearms, Injury, Readmissions, Injury severity

## Abstract

**Background:**

National conversation has justifiably been concerned with firearm-related deaths and much less attention has been paid to the consequences of surviving a firearm injury. We assessed the risk of hospital readmission, length of stay (LOS) during hospitalization, and costs within 90-days after surviving an index firearm injury and compared these data with pedestrians and occupants involved in motor vehicle crash (MVC).

**Methods:**

Nationwide Readmission Database, a nationally representative readmission database from 2013 and 2014 was used to create a retrospective cohort study. The primary outcome was time-to-first all-cause readmission within 90-days after discharge from the index hospitalization. Secondary outcomes were LOS and hospitalization costs at index events and at 90-days.

**Results:**

There were 3,334 (10.5%), 3,818 (10.6%) and 24,672 (9.4%) firearm injury, pedestrian, and occupant MVC readmissions within 90-days. The risk of 90-day readmission among firearm was 20% (HR=1.20, 95%CI=1.09-1.32) and 34% (HR=1.34, 95%CI=1.26-1.44) greater than patients admitted after pedestrian and occupant MVC. The primary causes of firearm readmission were surgical complications, intestinal disorders and open wounds. The mean total costs were lower among patients after firearm injury versus occupant MVC hospitalizations ($9,357 versus $11,032, p=0.028) but mean total LOS was greater (4.48 versus 4.38 days, p=0.003). Medicaid-insured patients had longer LOS at a total lower cost during index hospitalization after firearm injury as compared to MVC occupant injury. Increased LOS and lower costs of 90-day readmissions among firearm patients versus occupant MVC were irrespective of insurance.

**Conclusions:**

The patients surviving a firearm injury have a substantial risk of subsequent hospitalizations, higher than pedestrian or occupant MVC injuries. Medicaid is disproportionately burdened by the costs of treatment of firearm injury.

## Introduction

In the United States, firearm violence caused 440,095 deaths and an estimated 1,002,647 non-fatal injuries between 2001 and 2014.^1^ National estimates indicate that 40% of non-fatal firearm injuries are treated at an emergency room (ER) and released,^1^ while the remaining 60% are hospitalized after acute care in the ER.^2^ The costs of medical care, treatment, and recovery associated with firearm injury have increased enormously from 1994 to 2011.^3,4^ The economic burden associated with pediatric firearm hospitalization was estimated to be more than $1 billion between 2006 and 2009, and in adults from 2006 to 2010 was $88.6 billion, with an estimated average annual cost of $17.7 billion.^3,5^ These high costs were attributed to a longer hospital stay, need for rehabilitation after hospitalization, and the high in-hospital mortality based on the location and the severity of injuries sustained.^3,5,6^

Although there have been studies that have documented the outcomes of the index hospitalization for firearm injuries using national level^7,8^ and trauma center data,^6^ there are little data regarding the outcomes of firearm injuries that survive hospitalization for the initial firearm injury. In this context, the risk of subsequent readmission is a reasonable marker of ongoing injury severity for any injury that requires a first hospitalization.^9^ Therefore, in the present investigation we used nationally representative readmissions data for the years 2013 and 2014 to determine the risk of readmission, readmission related length of stay (LOS), and costs during the first 90-days after surviving an index hospitalization due to firearm injury. Given that the risk of subsequent mortality and morbidity is driven by the severity of injury, ^10^ we also assessed the risk of readmission while taking into consideration the levels of injury severity at the index injury. In order to place our findings in an appropriate and intuitive perspective, we chose to compare our findings on firearm injury to motor vehicle injury, which have been previously compared.^11,12^ In a motor vehicle crash, occupants are passengers in the motor vehicle, whereas pedestrians are those persons who are on the road and hit by a motor vehicle. We compared the risk of readmission among patients suffering firearm injuries to that for pedestrians and occupants involved in motor vehicle crashes (MVCs), building on prior studies.^13,14^

## Methods 

***Study design***

We conducted a claims-based, retrospective, cohort study comparing index hospitalizations of firearm injury with that of hospitalizations from pedestrians and occupants involved in MVC.

***Data source***

We used the 2013 and 2014 Nationwide Readmissions Database (NRD)^15^ that contains nationally representative information on hospital admissions with patient linkage numbers to track readmissions within a state. 

The inclusion and exclusion criteria are presented in **Supplementary Appendix 2 **. Firearm, pedestrian MVC and occupant MVC hospitalizations were identified using ICD-9-CM injury codes given in **S Table 1**. School of Medicine at Boston University institutional review board approved this study (H-35309).

***Study variables ***

The primary outcome was the time-to-first readmission within 90 days after discharge following an index hospitalization due to fire arm injury or a MVC. This included all fatal and non-fatal readmissions after index hospitalization discharge. The secondary outcomes are all-cause readmission at 30 and 60 days. The primary diagnosis of readmissions at 90-days was also categorized based on most frequent diagnoses using ICD-9 codes shown in**S Table 1**. Secondary outcomes were length of stay in days and hospitalization costs in US dollars ($). NRD provides total charges per hospitalization and the cost-to-charge conversion ratio, which was used to calculate costs.

We considered severity of injury primarily by using the New Injury Severity Score (NISS), measured using ICD-9 diagnostic codes and the ICD Programs for Injury Characteristic (ICDPIC), a Stata module that translates diagnosis codes into standard injury categories and scores.^16^ We also considered NISS categorized as quartiles.^17,18^ The other measure of injury severity based on location used was Injury Severity Score (ISS).^10,19,20^ We used only the primary NISS and ISS variables in the present analysis. The other covariates are detailed in**Supplementary Appendix 3**.

***Statistical analysis***

Two comparisons were performed: 1) firearm versus pedestrian MVC and 2) firearm versus occupant MVC. Severity of injury was calculated and compared using ICDPIC version 3.0 in STATA 14.2.^16^ We used survey-weighted Cox proportional hazards regression models, stratified by NISS to allow the baseline risk to vary by NISS, to determine the hazards ratio (HR), their 95% CI and the corresponding p values. All analyses were survey-weighted and were performed using STATA 14.2 (StataCorp LP, College Station, Texas; 2009) using weights provided in the datasets and using methodology specified by the distributor of the data. The detailed steps in statistical analysis are presented in **Supplementary Appendix 4**.

## Results

***Participants and follow up***

Patient and hospital characteristics are described in Table 1. Compared to patients injured as pedestrians and occupants in MVCs, patients injured by firearms were mostly men, young, had Medicaid insurance and were from neighborhoods with the median household income <$38,000. There were no differences between firearm and pedestrian MVC groups in the location of patient residences and the proportion of patients residing in the same area as the hospital at which they were treated, and the hospital characteristics, such as urban versus rural location. Almost half (47.4%) of the firearm group had no comorbidities, a larger proportion than both MVC groups (40.2%, 41.1% respectively).

**Table 1:** Baseline patient, hospital and injury characteristics.

	Firearm injury	MVC injury		P, Firearm vs.	
		Pedestrians	Occupants	Pedestrians	Occupants
n	31,610	36,164	262,906		
Year					
2013	15,775 (49.9)	18,368 (50.8)	134,676 (51.2)		
2014	15,835 (50.1)	17,796 (49.2)	128,230 (48.8)		
Age, mean (SE)	30.3 (0.2)	40.8 (0.4)	42.0 (0.2)	<0.0001	<0.0001
Age, n (%)				<0.0001	<0.0001
0-15	1,274 (4.0)	4,978 (13.8)	17,689 (6.7)		
16-24	12,093 (38.3)	5,578 (15.4)	49,706 (18.9)		
25-34	9,243 (29.2)	5,058 (14.0)	43,583 (16.6)		
35-44	4,339 (13.7)	4,200 (11.6)	34,243 (13.0)		
45-54	2,636 (8.3)	5,689 (15.7)	41,285 (15.7)		
55-90	2,026 (6.4)	10,661 (29.5)	76,401 (29.1)		
Gender				<0.0001	<0.0001
Men	28,068 (88.8)	24,133 (66.7)	169,646 (64.5)		
Women	3,543 (11.2)	12,031 (33.3)	93,260 (35.5)		
Location				0.21	<0.0001
Central Metro (>1m)	13,005 (41.4)	15,275 (42.9)	58,083 (22.2)		
Fringe Metro (>1m)	6,373 (20.3)	7,944 (22.3)	61,273 (23.4)		
Metro (250k-1m)	6,374 (20.3)	6,650 (18.7)	58,182 (22.2)		
Micropolitan	5,660 (18.0)	5,739 (16.1)	84,214 (32.2)		
Insurance				<0.0001	<0.0001
Private/Medicare	8,117 (25.7)	20,837 (57.9)	170,987 (65.4)		
Medicaid/Self/ No charge/other	23,420 (74.3)	15,177 (42.1)	90,560 (34.6)		
Median household income national quartile				<0.0001	<0.0001
$1-$37,999	16,995 (54.6)	12,270 (34.6)	72,444 (28.1)		
$38,000-$47,999	7,279 (23.4)	8,861 (25.0)	72,679 (28.2)		
$48,000-$63,999	4,658 (15.0)	7,685 (21.7)	63,276 (24.5)		
>=$64,000	2,199 (7.1)	6,645 (18.7)	49,671 (19.2)		
Patient resident same as hospital state	29,217 (92.4)	33,638 (93.0)	229,713 (87.4)	0.39	<0.0001
Hospital					
Bed size				0.43	0.068
Small	1,561 (4.9)	1,984 (5.5)	15,613 (5.9)		
Medium	7,018 (22.2)	7,226 (20.0)	48,133 (18.3)		
Large	23,031 (72.9)	26,954 (74.5)	199,160 (75.8)		
Teaching status				0.0005	<0.0001
Metro, non-teaching	4,013 (12.7)	5,831 (16.1)	54,186 (20.6)		
Metro, teaching	26,790 (84.8)	29,606 (81.9)	194,758 (74.1)		
Non-metro	807 (2.6)	728 (2.0)	13,963 (5.3)		
Urban hospital	20,600 (65.2)	24,680 (68.2)	130,543 (49.7)	0.13	<0.0001
Injury severity					
Computed new injury severity score (NISS), mean (SE)	13.8 (0.2)	12.9 (0.2)	13.5 (0.1)	<0.0001	0.070
Computed new injury severity score (NISS), n (%)				<0.0001	<0.0001
0-6 (1st quartile)	9232 (29.2)	10759 (29.8)	72333 (27.5)		
7-11 (2nd quartile)	7492 (23.7)	8117 (22.5)	57132 (21.7)		
12-17 (3rd quartile)	4592 (14.5)	9376 (25.9)	69244 (26.4)		
18-75 (4th quartile)	10292 (32.6)	7902 (21.9)	64009 (24.4)		
Injury severity scores (ISS) body region, n (%)				<0.0001	<0.0001
Head or neck	1990 (6.3)	9258 (25.6)	63494 (24.2)		
Face	1376 (4.4)	968 (2.7)	8365 (3.2)		
Chest	4014 (12.7)	3036 (8.4)	49248 (18.7)		
Abdominal or Pelvic contents	8032 (25.4)	2160 (6.0)	27209 (10.3)		
Extremities or pelvic girdle	11984 (37.9)	18497 (51.1)	102462 (39.0)		
External	3994 (12.6)	2046 (5.7)	11092 (4.2)		
Not further specified	219 (0.7)	199 (0.6)	1036 (0.4)		
Computed new injury severity score (NISS), mean (SE) in each ISS category					
Head or neck	20.3 (0.4)	15.4 (0.3)	15.2 (0.2)	<0.0001	<0.0001
Face	12.7 (0.5)	12.2 (0.4)	11.8 (0.2)	0.44	0.10
Chest	21.7 (0.4)	18.7 (0.4)	16.5 (0.2)	<0.0001	<0.0001
Abdominal or pelvic contents	18.6 (0.3)	14.9 (0.5)	13.8 (0.2)	<0.0001	<0.0001
Extremities or pelvic girdle	10.1 (0.1)	11.5 (0.1)	11.9 (0.1)	<0.0001	<0.0001
External	4.9 (0.2)	4.2 (0.2)	5.2 (0.1)	0.013	0.25
Elixhauser comorbidity score					
Mean (SE)	1.18 (0.01)	1.22 (0.02)	0.94 (0.02)	<0.0001	<0.0001
Categories, n (%)				<0.0001	<0.0001
0	14998 (47.4)	14524 (40.2)	108169 (41.1)		
1	8677 (27.4)	9572 (26.5)	70524 (26.8)		
2	4569 (14.5)	6096 (16.9)	43314 (16.5)		
>=3	3367 (10.7)	5972 (16.5)	40900 (15.6)		

All values are weighted frequency and percentages except first line of age, which is weighted mean and standard error. P-value is derived from chi-square test for all comparisons except comparison of mean and standard error of age, which was tested using survey-weighted linear regression.

The mean NISS score was 13.8 in the firearm group as compared to 12.9 and 13.5 in the pedestrian and occupant groups, respectively. There were 32.6% of firearm injury patients in the highest severity quartile category as compared to 21.9% and 24.4% among the pedestrian and occupant patients. The majority of firearm patients sustained primary injuries on their extremities (37.9%) and abdomen (25.4%), whereas pedestrian injuries were located in the extremities (51.1%) and head or neck (25.6%). The occupant injuries were also to a lesser degree located in the extremities (39.0%) with injuries also to the head or neck (24.2%) and the chest (18.9%). Location-specific NISS demonstrated head or neck injuries to be very severe among firearm injury patients (mean=20.3) as compared to 15.4 and 15.2 among pedestrians and occupants and similar gradients of injury severity for chest and abdominal injuries. The injury severity in the extremities was lowest among the firearm group (mean=10.1) as compared to the pedestrian (mean=11.5) and the occupant (mean=11.9) groups.

***Risk of injury severity***

The risk of overall injury severity using quartile categories of NISS for firearm versus pedestrian MVC and firearm versus occupant MVC demonstrates a U-shaped relationship (**S Table 2**). Firearm patients had a 33% and 22% increased likelihood of sustaining the highest versus lowest injury severity compared to pedestrians and occupants with MVC. Firearm patients had 47% and 49% lower likelihood for having an injury severity in the third quartile of NISS compared to pedestrians and occupants. Overall, this pattern was maintained after stratification by the location of primary injury comparing firearm injury patients with pedestrians or occupants with MVC (p for interaction<0.0001 for both). However, the likelihood of sustaining the highest severity of injury (4th quartile of NISS) to extremities in firearm injury patients was 21% and 38% lower compared to pedestrians and occupants involved in MVC.

***Risk of readmission ***

**Figure 1:**
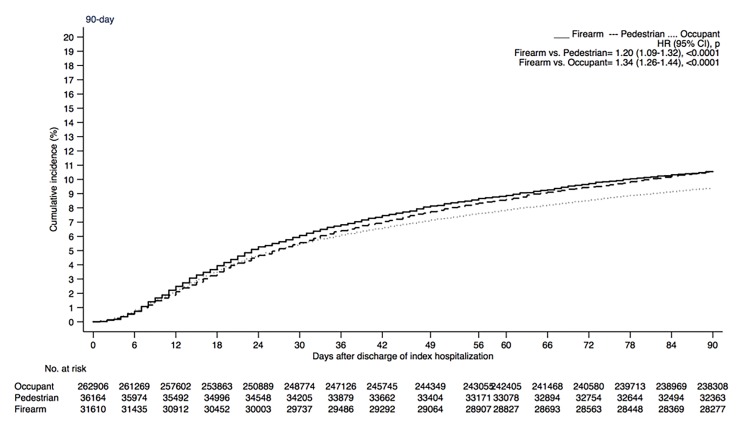
Kaplan Meier curves of risk of readmission after being discharged alive following firearm, pedestrian and occupant motor vehicle injury.

A total of 9,624 firearm- and MVC-injury patients had their first readmission within 3 months after discharge following the initial index event (Table 2). The first readmission absolute rates were 10.5%, 10.6% and 9.4% among firearm, pedestrian and occupant groups, respectively. At 90-days, the relative risk of readmission among firearm patients was 20% and 34% greater than pedestrians and occupants. At 30-days, the relative risk of readmission among patients with firearm injury was 26% and 34% greater than patients with MVC. At 60-days, the risk of readmission among firearm injury patients was 23% and 36% greater than pedestrians and occupants. The Kaplan Meier curves for readmission at 90 days following discharge after an index event are presented in Figure 1. The causes of first readmission within 90-days among patients with firearm injury were surgical complications (2.6%), intestinal disorders (0.9%) and open wounds (0.8%) (Table 3). Among the pedestrians, the top three causes were surgical complications (2.0%), fracture of lower limb (1.2%) and psychosis (0.9%), whereas after occupant MVC, the leading causes for readmission were surgical complications (1.3%), fracture of lower limb (1.3%) and cardiovascular and cerebrovascular diseases (0.6%).

**Table 2:** Risk of readmission at 30-days, 60-days and 90-days after surviving the injury.

	n (%)	Crude	P	Multivariable- 1		Multivariable- 2	
		HR (95% CI)		HR (95% CI)	P	HR (95% CI)	P
**At 30-days**							
Firearm	1917 (6.1)						
vs. Pedestrian	2009 (5.6)	1.10 (0.98-1.22)	0.10	1.20 (1.07-1.36)	0.003	1.26 (1.12-1.43)	<0.0001
vs. Occupant	14481 (5.5)	1.10 (1.01-1.20)	0.026	1.26 (1.15-1.39)	<0.0001	1.34 (1.22-1.47)	<0.0001
**At 60-days**							
Firearm	2804 (8.9)						
vs. Pedestrian	3110 (8.6)	1.04 (0.94-1.14)	0.48	1.17 (1.06-1.31)	0.003	1.23 (1.10-1.37)	<0.0001
vs. Occupant	20665 (7.9)	1.13 (1.05-1.22)	0.001	1.30 (1.20-1.41)	<0.0001	1.36 (1.25-1.47)	<0.0001
**At 90-days**							
Firearm	3334 (10.5)						
vs. Pedestrian	3818 (10.6)	1.00 (0.92-1.09)	0.96	1.15 (1.05-1.25)	0.002	1.20 (1.09-1.32)	<0.0001
vs. Occupant	24672 (9.4)	1.13 (1.06-1.20)	<0.0001	1.30 (1.22-1.39)	<0.0001	1.34 (1.26-1.44)	<0.0001

Survey weighted cox proportional hazard regression was used to estimate hazards ratios (HR), 95% confidence intervals (95% CI) and P. Multivariable model 1 was adjusted for year, age, sex, location, insurance, median household income national quartile, hospital size, hospital teaching status and Elixhauser comorbidity score. Multivariable model 2 is model 1 and stratified by new computed injury score (NISS).

**Table 3:** Primary diagnoses of readmission within first 90 days.

	Firearm injury, n (%)	Pedestrians, n (%)	Occupants, n (%)	Firearm vs. Pedestrian,HR (95% CI)	Firearm vs. Occupant,HR (95% CI)
**All readmissions, n**	**3334**	**3818**	**24672**	****	****
In-hospital death	* ()	46 (0.1)	357 (0.1)	()	()
Infection or sepsis	118 (0.4)	230 (0.6)	1321 (0.5)	1.02 (0.67-1.55)	1.19 (0.87-1.62)
Endocrine disorders	33 (0.1)	47 (0.1)	302 (0.1)	1.19 (0.52-2.75)	1.02 (0.51-2.04)
Fluid and electrolyte disorders	18 (0.1)	57 (0.2)	201 (0.1)	0.73 (0.30-1.77)	1.05 (0.45-2.45)
Anemia and blood disorders	27 (0.1)	31 (0.1)	261 (0.1)	0.91 (0.44-1.91)	1.12 (0.63-1.98)
Psychosis	170 (0.5)	337 (0.9)	885 (0.3)	0.66 (0.46-0.93)	1.44 (1.05-1.97)
Other mental health disorders	20 (0.1)	121 (0.3)	418 (0.2)	0.13 (0.06-0.27)	0.31 (0.15-0.62)
Nervous system disorders	96 (0.3)	98 (0.3)	749 (0.3)	1.18 (0.64-2.17)	1.20 (0.75-1.94)
Cardio and cerebrovascular disorders	123 (0.4)	220 (0.6)	2203 (0.8)	1.04 (0.68-1.58)	0.93 (0.68-1.27)
Aneurysm, embolism or thrombosis	132 (0.4)	114 (0.3)	690 (0.3)	1.50 (0.93-2.43)	1.84 (1.29-2.64)
Respiratory disorders	176 (0.6)	184 (0.5)	1539 (0.6)	1.12 (0.76-1.63)	1.13 (0.86-1.50)
Oral cavity and thorax	35 (0.1)	54 (0.2)	315 (0.1)	0.90 (0.30-2.73)	1.42 (0.75-2.70)
Abdominal disorders	128 (0.4)	99 (0.3)	651 (0.2)	2.53 (1.50-4.25)	1.91 (1.24-2.90)
Intestinal disorders	297 (0.9)	63 (0.2)	628 (0.2)	6.14 (3.48-10.8)	5.36 (4.00-7.18)
Genitourinary disorders	132 (0.4)	111 (0.3)	820 (0.3)	1.97 (1.18-3.31)	2.22 (1.49-3.29)
Skin and subcutaneous disorders	136 (0.4)	224 (0.6)	865 (0.3)	0.75 (0.49-1.12)	1.25 (0.89-1.75)
Musculoskeletal disorders	173 (0.5)	217 (0.6)	1414 (0.5)	1.01 (0.64-1.59)	1.08 (0.80-1.45)
Fracture, skull	63 (0.2)	70 (0.2)	380 (0.1)	1.60 (0.55-4.60)	1.38 (0.72-2.65)
Fracture, neck and trunk	17 (0.1)	74 (0.2)	1071 (0.4)	0.40 (0.17-0.97)	0.18 (0.08-0.39)
Fracture, upper limb	68 (0.2)	77 (0.2)	649 (0.2)	1.77 (0.87-3.63)	1.35 (0.85-2.15)
Fracture, lower limb	139 (0.4)	432 (1.2)	3536 (1.3)	0.47 (0.30-0.72)	0.42 (0.32-0.57)
Fracture, intracranial	* ()	96 (0.3)	628 (0.2)	()	()
Internal injury	103 (0.3)	47 (0.1)	850 (0.3)	1.28 (0.68-2.41)	1.09 (0.71-1.68)
Open wounds	245 (0.8)	120 (0.3)	522 (0.2)	3.04 (1.87-4.92)	3.66 (2.54-5.27)
Nerve and spinal cord injury	* ()	* ()	48 (<0.1)	()	()
Iatrogenic	24 (0.1)	61 (0.2)	297 (0.1)	0.38 (0.15-0.97)	0.48 (0.21-1.11)
Surgical complications	828 (2.6)	720 (2.0)	3510 (1.3)	1.43 (1.11-1.83)	2.01 (1.71-2.38)
Medical devices and Rehabilitation	159 (0.5)	165 (0.5)	788 (0.3)	1.66 (1.02-2.71)	2.01 (1.39-2.91)

* All values are weighted. Clinical outcomes are derived from ICD-9 CM code indicating primary diagnosis from hospitalization after the index injury hospitalization. The events may not add up to total hospitalizations, since only the relevant events were individually represented. HR= hazards ratios.

***Stratified analysis ***

**Figure 2:**
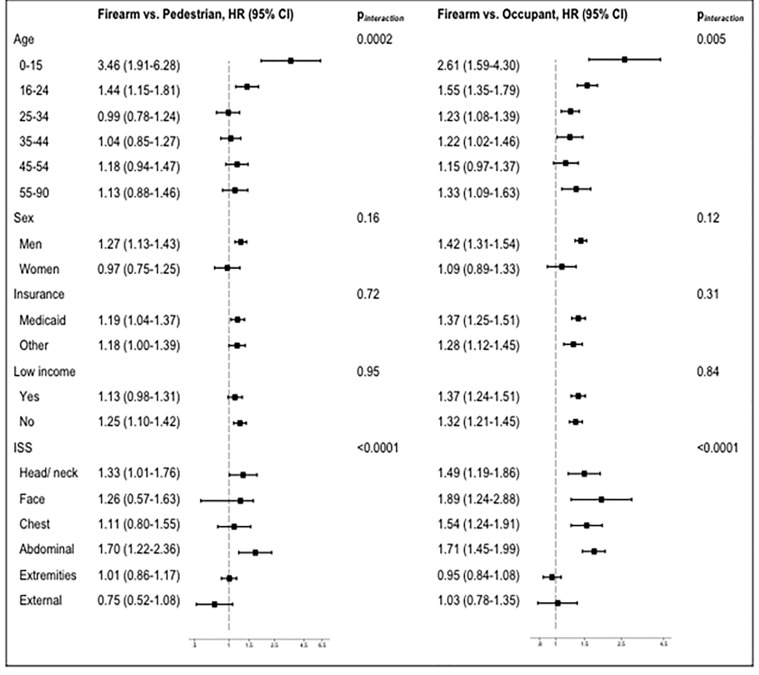
Stratified analysis of readmission within 90-days.

The stratified analysis of readmission within 90-days is presented in Figure 2. We observed effect modification by age when comparing the risk of readmission between the three groups. The risk of readmission among children below 15 years with a firearm injury was 3.5-times greater than pedestrians (p-interaction=0.002), and 2.6-times greater than occupants (p-interaction=0.005). Risk of readmission was 70% and 71% greater in firearm injury, compared to pedestrian and occupant MVC injury for abdominal injuries (p-interaction for injury location <0.0001). 

***Length of stay and cost of hospitalization***

Means and 95% confidence intervals (95% CI) predicted from multivariable analysis to assess differences in mean of total LOS, total costs and costs per readmission are presented in Table 4. The mean LOS and cost of treating index firearm injury hospitalization (3.71 days, $15,104) was lower than pedestrian injuries (4.13 days, $16,212, difference: -0.42 days, p=0.007; -$1,108, p=0.001), and greater than occupant injuries (3.38 days, $13,464, difference: 0.33 days, p<0.0001; $1,640, p<0.0001). At 90 days, the mean total LOS and total cost of treating firearm injury (index hospitalization and readmissions) (4.48 days, $9,357) was similar to pedestrian injuries (5.25 days, $11,463, difference: -0.77 days, p=0.41; -$2,106, p=0.14) and greater than occupant injuries (4.38 days, $11,038, difference: 0.10, p=0.003; $1,681, p=0.028). In the post-hoc stratified analysis by insurance in **S Table 3**, during the index hospitalization, there was no difference in LOS and total costs between those with Medicaid and those with private or Medicare insurance in firearm versus pedestrian injury patients. The increased mean total LOS among firearm versus occupant injury patients was greater in patients with Medicaid (difference: 0.40 days, p <0.0001) compared to private or Medicare insurance (difference: 0.23 days, p=0.048) (p-interaction=0.005). Among those who survived the acute hospitalization, after 90 days, there were no significant difference between firearm and occupant or pedestrian injury patients in the mean of total LOS, LOS per readmission, total costs and costs per readmission. 

**Table 4:** Multivariable association between injury types with costs and duration of index hospitalization and readmissions.

	Firearm injury	Pedestrian MVC		Occupant MVC	
	Mean (95% CI)	Mean (95% CI)	Difference, p	Mean (95% CI)	Difference, p
**Index injury**					
All, n	34,390	37,742		268,885	
Total cost	15104 (13898-16309)	16212 (14850-17574)	-1108, 0.001	13464 (12651-14277)	1640,<0.0001
Hospital duration in days	3.71 (3.42-4.03)	4.13 (3.79-4.47)	-0.42, 0.007	3.38 (3.20-3.55)	0.33, <0.0001
**30-days**					
All, n	1,917	2,009		14,481	
# of readmissions	1.05 (0.92-1.18)	1.05 (0.91-1.19)	0.0, 0.69	1.04 (0.96-1.11)	0.01, 0.95
Total cost	8281 (6518-10044)	10207 (7940-12473)	-1926, 0.036	10079 (8883-11275)	-1798, 0.036
Cost per readmission	7980 (6298-9661)	9793 (7634-11952)	-1813, 0.079	9751 (8613-10890)	-1771, 0.031
Hospital duration in days					
Total	4.08 (3.26-4.90)	4.47 (3.53-5.41)	-0.39, 0.68	3.82 (3.42-4.23)	0.26, 0.001
Per readmission	1.38 (1.21-1.54)	1.41 (1.23-1.59)	-0.03, 0.29	1.33 (1.25-1.42)	0.05, 0.015
**60-days**					
All, n	2,804	3,110		20,665	
# of readmissions	1.10 (0.95-1.23)	1.16 (1.01-1.24)	-0.06, 0.83	1.12 (1.04-1.19)	-0.02, 0.57
Total cost	9013 (7352-10675)	11265 (9136-13393)	-2252, 0.079	10694 (9611-11777)	-1681, 0.059
Cost per readmission	8313 (6845-9782)	10156 (8302-12009)	-1843, 0.16	9868 (8899-10836)	-1555, 0.038
Hospital duration in days					
Total	4.43 (3.61-5.25)	5.05 (4.11-6.00)	-0.62, 0.80	4.21 (3.81-4.61)	0.22, 0.001
Per readmission	1.37 (1.23-1.50)	1.42 (1.27-1.56)	-0.10, 0.80	1.33 (1.26-1.40)	0.04, 0.011
**90-days**					
All, n	3,334	3,818		24,672	
# of readmissions	1.17 (1.03-1.32)	1.23 (1.08-1.39)	-0.06, 0.83	1.18 (1.10-1.26)	-0.01, 0.80
Total cost	9357 (7750-1096)	11463 (9458-13468)	-2106, 0.14	11038 (9995-12080)	-1681, 0.028
Cost per readmission	8373 (7027-9719)	9954 (8312-11595)	-1581, 0.32	9832 (8950-10714)	-1459, 0.014
Hospital duration in days					
Total	4.48 (3.71-5.24)	5.25 (4.36-6.15)	-0.77, 0.41	4.38 (3.99-4.76)	0.10, 0.003
Per readmission	1.32 (1.20-1.45)	1.39 (1.25-1.52)	-0.07, 0.86	1.31 (1.24-1.37)	0.01, 0.083

All mean and 95% confidence intervals (95% CI) are survey-weighted. Since the distribution of cost and length of hospital stay is right-skewed, both were log transformed. Survey linear regression was used for cost and survey poisson regression for number of readmissions; mean and standard error (SE) was predicted from the model. Number of readmissions considered only those who had a readmission during the specific time period. The multivariable model was adjusted for year, age categories, county population, insurance payer, household income categories, hospital bedsize, hospital rural and teaching status, Elixhauser comorbidity score categories and intent of injury stratified by NISS. Difference is the difference between firearm versus pedestrian and firearm versus occupant. P-value is derived from the model.

## Discussion

Our study presents four main findings. First, those surviving an index firearm injury hospitalization have different degrees of injury severity, and the severity of injury depends on the site of injury. Second, firearm injury patients have an increased risk of readmission within 90-days after surviving index hospitalization compared to pedestrians and occupants involved in MVC. Third, children have the greatest risk of readmission during the first 90-days after discharge following the initial injury compared to adults of all age groups, and those with head or neck, facial, chest and abdominal injuries carry a higher risk of readmission during the first 90-days. On the other hand, the risk of readmission was similar irrespective of sex, neighborhood income level or insurance. Fourth, the increased LOS during index hospitalization in firearm versus occupant patients was significantly greater in Medicaid than private or Medicare insured patients, with no differential for increased cost. As for 90-day readmissions, Medicaid insured patients had similar LOS and costs of hospitalization as private or Medicare insured patients.

We demonstrated that a common profile for a patient hospitalized for firearm injury is a young man from a poor neighborhood; this is consistent with the findings from earlier studies that have assessed both fatal and non-fatal firearm injuries.^7,21^ Of note, non-fatal firearm injuries have been on the rise nationally since 2001, while fatal injuries have remained constant.^1,22^ This indicates a rising burden of injury particularly for young men living in economically disadvantaged settings, who are already marginalized and at high risk of poor health.

We demonstrated that the severity of injury sustained in the firearm group is greater than that in the MVC groups, and, in particular, this increased severity varied according to the location of the injury. The most severe firearm injuries were those to the head and neck, chest and abdomen. These location-specific effects of injury severity have been observed in other studies.^23,24^ In a 10-year retrospective cohort study of interpersonal violence victims, the risk of greater injury severity was shown to be from injuries to the head as compared to other parts of the body.^24^ We observed a U-shaped pattern of risk of increased injury severity when firearm injuries were compared with MVC using the NISS. These findings may suggest that the NISS may not be adequately capturing the severity of firearm injury. A retrospective study using the Singapore National Trauma Registry among those 18 years and older also suggested that the NISS may not be adequately capturing full morbidity for severe injury, and suggested that the NISS may be best used with additional attributes, such as markers of anatomical polytrauma injury.^25^ Another study that used the NISS found that among trauma center patients about half of those with highest scores indicative of “unsurvivable” injuries survived, also raising the question of the overall utility of the NISS as a single measure.^26^ Overall, these findings suggest that the NISS and injury severity measures may be useful for general quantification of injury severity, but may fall short for specific injury types such as firearm injuries.

Our central finding-that there is a greater likelihood of readmission during first 90-days among firearm injury survivors compared to MVC-is corroborated by previous reports using single center studies where firearm injury hospitalizations had a higher rate of in-hospital deaths at 8.1%^7^ as compared to <1% for MVC, although the outcomes are not exactly similar.^27^ The higher readmission risk among firearm injury survivors may extend beyond 90 days, and firearm injuries may be associated with higher rates of long-term health consequences.. Until now attention has focused largely on the outcomes related to firearm injury-related hospitalizations such as repeat victimization; our study reports that firearm injury can have clinical consequences.^28^ The acute consequences of MVC have been shown to have relatively limited long-term adverse outcomes for all but the most severely injured, consistent with our findings here.^29,30^

Children had a high risk of readmission during first 90-days: 356% greater than children with pedestrian MVC, and 261% greater than children with occupant MVC. While available evidence of an increased risk of violence perpetration among adults^28,31^ has led to a relative paucity of firearm injury, the increased risk among children observed by us establishes the relevance of burden of disease research among survivors of firearm violence. The increased readmission risk during the first 90-days for those with head or neck, facial, chest and abdominal injuries clearly shows that patients with certain critical injury locations carry an extended burden of disease even after surviving the acute phase of injury.

In our study, patients hospitalized for firearm injury are mostly younger men from poor neighborhoods, and three-fourths are insured by Medicaid. Compared to occupant MVC patients, Medicaid-insured firearm patients stayed an additional 0.40 days in the hospital while the private or Medicare insured stayed an additional 0.23 days. However, the increased LOS among firearm patients as compared to occupant MVC were not echoed in hospitalization costs. There were no differences in firearm versus occupant MVC patients who had Medicaid compared to private insurance or Medicare. Furthermore, when considering the total burden of hospitalization during the first 90-days after surviving the injury, there were no differences in LOS or costs between the three groups by insurance types. This observation suggests that Medicaid provided longer stays during the index injury and provided comparable care to the private or Medicare insurance during subsequent visits. Our results are in line with several value-based health care programs that are implemented in Medicaid that demonstrate cost-savings despite longer hospitalization duration compared to private or Medicare insurance.^32,33^

Our results have to be interpreted in the light of some limitations. First, we are using data from claims-based hospitalizations, where we do not have active follow-up to assess mortality or other non-hospitalization morbidity after being discharged alive. Second, the lack of longer follow up restricts our analysis, preventing longer term follow up analysis approaches that might be useful to address the questions at hand. Third, although the patient-level data is weighted to allow for national estimates, the sample does not provide race/ethnicity variable, which precludes analysis to explore race/ethnicity differences in risk. Fourth, the data collection procedures may have been different in different hospitals and states, which may result in possible misclassification bias. Injuries are captured as secondary diagnosis, which in turn does not allow accurate identification of new firearm injury or recidivism. On the other hand, the lack of sufficient follow up duration after surviving the index hospitalization may have underestimated the counts of readmissions, which we have attempted to correct by excluding the index injuries after September. Fifth, we were unable to assess the state-specific differences in injury type due to the lack of state-specific information. 

In summary, patients surviving an initial firearm injury have substantial continuing morbidity following their survival of the acute phase and after discharge, more so than do comparable MVC injury survivors. This underscores a public health problem attributable to the health consequences of firearms in the US and the need for additional research on firearm survivorship focusing on these outcomes. Additionally, our study suggests that Medicaid insurance provides much of the acute hospitalization care and continuing survivorship care to the firearm injury patients.^22^

**Acknowledgment**

We thank Elizabeth Pino, PhD, Center for Clinical Translational Epidemiology and Comparative Effectiveness Research, Department of Medicine, School of Medicine, Boston University for editing the manuscript.

**SUPPLEMENT**

**Risk of 90-day readmission among firearm injury hospitalization: A nationally representative retrospective cohort study.**

**Supplementary Table 1:** ICD-9 diagnostic codes used for categorizing firearm, pedestrian motor vehicle and occupant motor vehicle injuries and for categorizing primary diagnosis of readmissions.

**Firearm injury**	****
Unintentional or accident	E9220, E9221, E9222, E9223, E9224, E9228, E9229
Assault	E9650, E9651, E9652, E9653, E9654
Suicide	E9550, E9551, E9552, E9553, E9554, E9556, E9559
Legal	E970
War	E991
Undetermined	E9850, E9851, E9852, E9853, E9854, E9856
**Motor Vehicle injuries**	****
Pedestrian	E8106, E8107,
	E8116, E8117,
	E8126, E8127,
	E8136, E8137,
	E8146, E8147,
	E8156, E8157,
	E8166, E8167,
	E8176, E8177,
	E8186, E8187,
	E8196, E8197,
	E8206, E8207,
	E8216, E8217,
	E8226, E8227,
	E8236, E8237,
	E8246, E8247,
	E8256, E8257
Occupant	E8100, E8101, E8102, E8103, E8104, E8105, E8108,
	E8110, E8111, E8112, E8113, E8114, E8115, E8118,
	E8120, E8121, E8122, E8123, E8124, E8125, E8128,
	E8130, E8131, E8132, E8133, E8134, E8135, E8138,
	E8140, E8141, E8142, E8143, E8144, E8145, E8148,
	E8150, E8151, E8152, E8153, E8154, E8155, E8158,
	E8160, E8161, E8162, E8163, E8164, E8165, E8168,
	E8170, E8171, E8172, E8173, E8174, E8175, E8178,
	E8180, E8181, E8182, E8183, E8184, E8185, E8188,
	E8190, E8191, E8192, E8193, E8194, E8195, E8198,
	E8200, E8201, E8202, E8203, E8204, E8205, E8208,
	E8210, E8211, E8212, E8213, E8214, E8215, E8218,
	E8220, E8221, E8222, E8223, E8224, E8225, E8228,
	E8230, E8231, E8232, E8233, E8234, E8235, E8238,
	E8240, E8241, E8242, E8243, E8244, E8245, E8248,
	E8250, E8251, E8252, E8253, E8254, E8255, E8258,
Infections	001x-139x
Endocrine disorders	24x-25x
Fluid and electrolyte disorders	276x
Anemia and blood disorders	28x
Psychosis	29x
Other mental health disorders	30x, 31x
Nervous system disorders	32x, 33x, 34x, 35x, 36x, 37x, 38x
Cardio and cerebrovascular disorders	40x, 41x, 42x, 43x
Aneurysm, embolism or thrombosis	44x, 45x
Respiratory disorders	46x, 47x, 48x, 49x, 50x, 51x
Oral cavity and thorax	52x, 53x
Abdominal disorders	55x, 57x
Intestinal disorders	56x
Genitourinary disorders	58x, 59x, 60x, 61x, 62x
Skin and subcutaneous disorders	68x, 69x, 70x
Musculoskeletal disorders	71x, 72x, 73x
Fracture, skull	800x, 801x, 802x, 803x, 804x
Fracture, neck and trunk	805x, 806x, 807x, 808x, 809x
Fracture, upper limb	81x
Fracture, lower limb	82x
Fracture, intracranial	85x
Internal injury	86x
Open wounds	87x, 88x, 89x
Nerve and spinal cord injury	950x, 951x, 952x, 953x, 954x, 955x, 956x, 957x
Iatrogenic	96x, 97x, 98x
Surgical complications	996x, 997x, 998x, 999x
Medical devices and Rehabilitation	V5x

**Supplementary Table 2:** Risk of injury severity associated with type of injury.

	Quartiles of computed new injury severity, OR (95%CI)				p	p-interaction
	0-6	7-11	12-17	18-75		
Firearm vs. Pedestrian MVA						<0.0001
Overall	Reference	1.02 (0.93-1.12)	0.53 (0.47-0.59)	1.33 (1.20-1.47)	<0.0001	
Head/ neck	Reference	2.18 (1.49-3.18)	1.88 (1.28-2.75)	3.09 (2.15-4.43)	<0.0001	
Face	Reference	0.97 (0.62-1.51)	0.65 (0.43-0.99)	1.28 (0.80-2.04)	0.019	
Chest	Reference	1.57 (1.00-2.45)	0.74 (0.47-1.17)	2.55 (1.76-3.68)	<0.0001	
Abdominal/ pelvic	Reference	1.18 (0.87-1.62)	0.86 (0.64-1.17)	2.53 (1.96-3.26)	<0.0001	
Extremities/ pelvic girdle	Reference	1.50 (1.34-1.69)	0.53 (0.45-0.61)	0.79 (0.68-0.93)	<0.0001	
External	Reference	1.44 (0.93-2.24)	1.04 (0.66-1.63)	1.90 (1.03-3.51)	0.096	
Firearm vs. Occupant MVA						<0.0001
Overall	Reference	1.04 (0.97-1.11)	0.51 (0.48-0.56)	1.22 (1.12-1.31)	<0.0001	
Head/ neck	Reference	2.74 (1.93-3.90)	2.33 (1.65-3.30)	4.14 (2.97-5.75)	<0.0001	
Face	Reference	0.80 (0.57-1.11)	0.61 (0.46-0.81)	1.29 (0.93-1.79)	<0.0001	
Chest	Reference	2.04 (1.51-2.77)	0.94 (0.69-1.29)	4.63 (3.57-6.00)	<0.0001	
Abdominal/ pelvic	Reference	1.07 (0.88-1.30)	1.02 (0.86-1.20)	3.04 (2.65-3.50)	<0.0001	
Extremities/ pelvic girdle	Reference	1.38 (1.26-1.51)	0.49 (0.44-0.55)	0.62 (0.54-0.70)	<0.0001	
External	Reference	1.21 (0.94-1.56)	0.88 (0.66-1.18)	1.24 (0.89-1.74)	0.20	

Survey weighted multinomial logistic regression was used to estimate odds ratios (OR) and 95% confidence intervals (95% CI).All models are multivariable and adjusted for year, age, sex, location, insurance, median household income national quartile, hospital size, hospital teaching status and Elixhauser comorbidity score.

**Supplementary Table 3:** Stratified analysis by insurance type in the relation between injury types with costs and duration of index hospitalization and readmis-sions [Post-hoc analysis].

	Firearm injury	Pedestrian MVC			Occupant MVC		
	Mean (95% CI)	Mean (95% CI)	Diff, p	p-inter	Mean (95% CI)	Diff, p	p-inter
**Index injury**							
All, n	34,390	37,742			268,885		
Medicaid, n	25,063	15,761			92,296		
Private/Medicare, n	9,247	21,821			175,180		
Total cost, all	15104 (615)	16212 (695)	-1108, 0.001	0.28	13464 (415)	1640, <0.0001	0.78
Medicaid	15287 (722)	17484 (927)	-2197, 0.001		14012 (529)	1275, <0.0001	
Private/Medicare	14674 (771)	15327 (765)	-653, 0.035		13178 (424)	1496, 0.028	
Hospital duration in days, all	3.71 (0.16)	4.13 (0.17)	-0.42, 0.007	0.051	3.38 (0.09)	0.33, <0.0001	0.005
Medicaid	3.76 (0.20)	4.26 (0.24)	-0.50, 0.44		3.36 (0.12)	0.40, <0.0001	
Private/Medicare	3.62 (0.20)	4.03 (0.20)	-0.41, 0.001		3.39 (0.09)	0.23, 0.048	
**90-days (with readmissions)**							
All, n	3,331	3,812			24,571		
Medicaid, n	2,425 (72.7%)	1,549 (40.6%)			7,706 (31.4%)		
Private/Medicare, n	906 (27.2%)	2263 (59.4%)			16,865 (68.6%)		
# of readmissions, all	1.17 (0.08)	1.23 (0.08)	-0.06, 0.83	0.70	1.18 (0.04)	-0.01, 0.80	0.98
Medicaid, n	1.17 (0.10)	1.23 (0.11)	-.0.06, 0.52		1.18 (0.07)	-0.01, 0.41	
Private/Medicare, n	1.18 (0.12)	1.24 (0.11)	-0.06, 0.85		1.18 (0.05)	0.0, 0.92	
Total cost, all	9357 (820)	11463 (1023)	-2106, 0.14	0.31	11038 (532)	-1681, 0.028	0.65
Medicaid, n	9001 (1005)	11152 (1427)	-2151, 0.16		10430 (828)	1429, 0.050	
Private/Medicare, n	10399 (1374)	11723 (1353)	1324, 0.57		11310 (637)	-911, 0.37	
Cost per readmission, all	8373 (687)	9954 (837)	-1581, 0.32	0.19	9832 (450)	1459, 0.014	0.56
Medicaid, n	8029 (841)	9661 (1170)	-1632, 0.19		9284 (700)	-1255, 0.031	
Private/Medicare, n	9376 (1166)	10183 (1107)	-807, 0.93		10081 (540)	-705, 0.37	
Total LOS, all	4.48 (0.39)	5.25 (0.46)	-0.77, 0.41	0.26	4.38 (0.20)	0.10, 0.003	0.55
Medicaid, n	4.36 (0.48)	5.22 (0.66)	-0.86, 0.46		4.18 (0.31)	0.18, 0.025	
Private/Medicare, n	4.87 (0.63)	5.30 (0.61)	-0.43, 0.96		4.47 (0.23)	0.40, 0.029	
LOS Per readmission, all	1.32 (0.06)	1.39 (0.07)	-0.07, 0.86	0.21	1.31 (0.03)	0.01, 0.083	0.49
Medicaid, n	1.31 (0.08)	1.38 (0.10)	-0.07, 0.64		1.28 (0.05)	0.03, 0.17	
Private/Medicare, n	1.37 (0.10)	1.39 (0.09)	-0.02, 0.55		1.32 (0.04)	0.05, 0.15	

All mean and standard error are weighted. Since the distribution of cost and length of hospital stay is right-skewed, both were log transformed. Survey linear regression was used for cost and survey poisson regression for number of readmissions; mean and standard error (SE) was predicted from the model. Number of readmissions considered only those who had a readmission during the specific time period. The multivariable model was adjusted for year, age categories, county population, insurance payer, household income categories, hospital bedsize, hospital rural and teaching status, Elixhauser comorbidity score categories and intent of injury stratified by NISS. Difference is the difference between firearm versus pedestrian and firearm versus occupant. P-value is derived from the model and 1P value are for interaction between the two stratum.

**Supplementary Figure 1:**
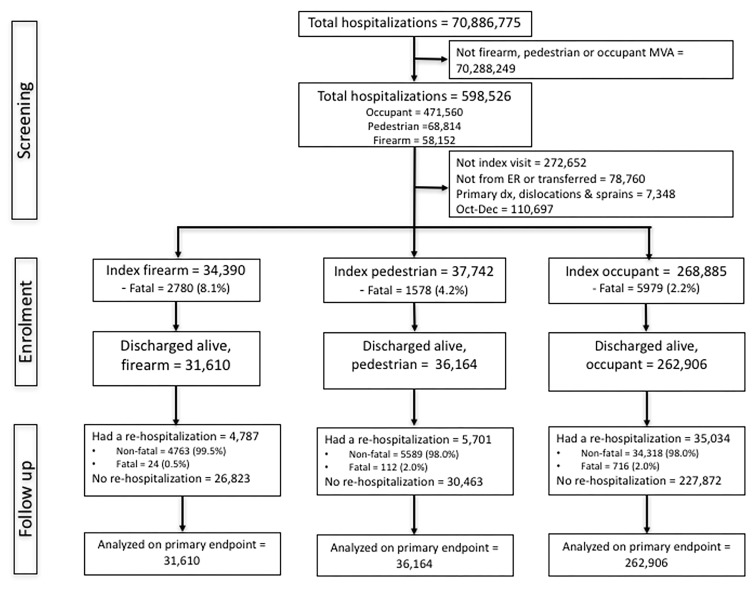
Flowchart.

**Supplementary Appendix 1: Details of 2013 and 2014 Nationwide Readmissions Database**

The detailed account of data source is presented in **Supplementary Appendix 1**. In the 2013 NRD, there are approximately 14 million discharges from 2,006 hospitals from 21 state inpatient databases; representing 49.3% of the US population and 49.1% of US hospitalizations. In the 2014 NRD, there are approximately 14 million discharges from 2,048 hospitals from 22 state inpatient databases; representing 51.2% of the US population and 49.3% of US hospitalizations. The NRD includes all discharges and those who have died in the hospital. Diagnoses and procedures during each hospitalization are categorized using the International Classification of Dis-eases, Ninth Revision, Clinical Modification (ICD-9-CM) codes. The readmissions within a year could be identified but data are not designed to be linked across years. Therefore, we used discharges during the first nine months of 2013 and 2014 to allow a minimum follow up duration of 3 months after an index event, i.e., initial hospitalization following a firearm-related injury. The data are available for purchase from https://www.hcup-us.ahrq.gov/tech_assist/centdist.jsp. 

**Supplementary Appendix 2: Inclusion and exclusion criteria**

We identified all firearm, pedestrian MVC and occupant MVC hospitalizations using ICD-9-CM injury codes given in **S Table 1**. First, we identified all the first visits for each of the three groups. From 70,886,775 weighted hospitalizations, we identified 598,526 potential visits belonging to each of these groups. Second, we excluded those hospitalizations that were not index visits (n=272,652), that were not explicitly admitted from the ER, or were transferred from another hospital (n=78,760), those where the primary diagnosis was injury but was dislocations and sprains (n=7,348), indicative of non-index injury, but a subsequent hospitalization and those index events during October, November and December in both years to allow at least 3 months of follow up. These exclusions were performed to minimize selection bias if the hospitalization was a repeat hospitalization after a prior injury. There were 34,390, 37,742 and 268,885 firearm, pedestrian MVC and occupant MVC index hospitalizations. A total of 10,337 died [2,780 (8.1%), 1,578 (4.2%) and 5979 (2.2%)] during their index hospitalizations in the respective groups. The remaining 31,610, 36,164 and 268,885 were included in our study. The flow chart for patient selection is shown in S Figure 1.

**Supplementary Appendix 3: Covariates**

The patient-level covariates used were age (categories of 0-15, 16-24, 25-34, 35-44, 45-54 and 55-90), sex (men and women), location (cen-tral metro with > 1 million population, fringe metro with > 1 million population, metro with population 250,000 to <1 million and micropolitan areas), insurance provider (private/ Medicare and Medicaid/ self-pay/ no charge/ other forms), median household national income quartiles ($1-$37,999, $38,000-$47,999, $48,000-$63,999 and >=$64,000), and whether the patient resided in the same state as the hospital. We also used the clinical comorbidities at the index hospitalization already derived in the dataset from ICD-9 diagnosis codes, and assessed cumulative comorbidity using the Elixhauser comorbidity score. ^14^

Hospital-level covariates were bed size of the hospital (small, medium and large), teaching status of the hospital (metro non-teaching, metro teaching and non-metro) and whether the hospital was an urban hospital. Description of data elements in the data are described in https://www.hcup-us.ahrq.gov/db/nation/nrd/nrddde.jsp.

**Supplementary Appendix 4: Detailed steps in statistical analysis**

First, we compared the baseline patient, hospital and injury characteristics; categorical variables were compared using chi-square tests and con-tinuous variables were compared using the Student’s t-test. 

Second, we assessed the risk of injury severity (four categories of NISS and locations of injury using ISS) associated in two comparisons, using survey-weighted multinomial logistic regression to determine the odds ratios (OR), their 95% confidence intervals (95% CI) and the corresponding p values. 

Third, we used survey-weighted Cox proportional hazards regression models, stratified by NISS to allow the baseline risk to vary by NISS, to determine the hazards ratio (HR), their 95% CI and the corresponding p values. The multivariable model was adjusted for age, sex, location, insur-ance, median household income national quartile, hospital size, hospital teaching status and Elixhauser comorbidity score. 

Fourth, Kaplan Meier curves using weighted survey estimates were constructed after truncating at 90-days of follow up. Those patients who did not have a readmission until the end of each year were assumed to be alive until the end of that year. 

Fifth, we explored the primary diagnosis of each readmission, categorized it based on the most frequent diagnosis, and estimated the HR and 95% CI using survival analysis for each relevant category of readmission. 

Sixth, the effect modification by age groups, sex, comorbidities and location of injury was also assessed along with p for interaction by incor-porating a multiplicative term between the effect modifier and injury groups into the models. 

Seventh, survey weighted Poisson regression and linear regression of log transformed number of readmissions, LOS and costs of hospitaliza-tion was performed and the mean and standard error (SE) was predicted from the adjusted model in the three groups and compared. We also performed a post-hoc stratified analysis by insurance. 
